# Assessing the impact of a novel house design on the incidence of malaria in children in rural Africa: study protocol for a household-cluster randomized controlled superiority trial

**DOI:** 10.1186/s13063-022-06461-z

**Published:** 2022-06-20

**Authors:** Salum Mshamu, Arnold Mmbando, Judith Meta, John Bradley, Thomas Chavalier Bøjstrup, Nicholas P. J. Day, Mavuto Mukaka, Fredros Okumu, Ally Olotu, Christopher Pell, Jacqueline Deen, Jakob Knudsen, Steven W. Lindsay, Lorenz von Seidlein

**Affiliations:** 1 CSK Research Solutions, Mtwara, Tanzania; 2 Nuffield Department of Clinical Medicine, University of Oxford, Oxford, UK; 3 Ifakara Health Institute, Ifakara, Tanzania; 4 Department of Biosciences, Durham University, Durham, UK; 5 University of Amsterdam, Amsterdam, Netherlands; 6 MRC International Statistics and Epidemiology Group, London School of Hygiene and Tropical Medicine, London, UK; 7 The Royal Danish Academy of Fine Arts, Copenhagen, Denmark; 8 Mahidol-Oxford Tropical Medicine Research Unit (MORU), Bangkok, Thailand; 9 University of Philippines, Manila, Philippines

**Keywords:** Housing, House screening, Malaria, Respiratory infections, Diarrhoea, Tanzania, Africa

## Abstract

**Background:**

Traditional rural housing in hot, humid regions of sub-Saharan Africa usually consists of single-level, poorly ventilated dwellings. Houses are mostly poorly screened against malaria mosquitoes and limited airflow discourages the use of bednets resulting in high indoor transmission. This study aims to determine whether living in a novel design house with elevated bedrooms and permeable screened walls reduces malaria, respiratory tract infections, and diarrhoea among children in rural Tanzania.

**Methods/study design:**

This is a household-randomized, controlled study in 60 villages in Mtwara, Tanzania. A total of 550 households are randomly selected, 110 of which are allocated a novel design house and 440 households continue to reside in traditional houses. A dynamic cohort of about 1650 children under 13 years will be enrolled and followed for 3 years, approximately 330 living in novel design houses and 1320 in traditional rural houses. The primary endpoint is the incidence of malaria; secondary endpoints are incidences of acute respiratory tract infections and diarrhoea diseases detected by passive and active surveillance. Exposure to malaria vectors will be assessed using light traps in all study houses. Structural, economic, and social science studies will assess the durability, cost-effectiveness, and acceptability of the new houses compared with traditional housing. Environmental data will be collected indoors and outdoors in study homes to assess the differences between house typologies.

**Discussion:**

This is the first randomized controlled trial to assess the protective efficacy of a new house design targeting malaria in sub-Saharan Africa. The findings of this study could influence the future construction of homes in hot and humid zones of Africa.

**Trial registration:**

ClinicalTrials.govNCT04529434. Registered on August 27, 2020

## Background

The population of Africa, currently around 1.3 billion, is projected to double by 2050 and reach more than 4 billion by 2100 [1]. This population growth will create demand for healthier, more comfortable, and more affordable homes. Sustainable Development Goal 11 proposes adequate, safe, and affordable housing for everyone by 2030 [2]. To achieve this goal, it will be important to know what constitutes a healthy living space.

Traditional rural house construction in hot and humid regions of sub-Saharan Africa often consists of wattle and daub with a thatched roof resulting in single-level, poorly ventilated spaces which can place children at risk of a range of diseases such as malaria, respiratory tract infections, and diarrhoea [3]. Walls constructed of heavy materials with small or absent windows result in an uncomfortable indoor climate that is hot during the night and even hotter during the day. These conditions hamper the use of insecticide-treated nets (ITNs), which further attenuates airflow by a mean of 60% [4, 5]. Compared to elevated bedrooms, single-storey buildings harbour higher mosquito densities [6]. Recently, it has been demonstrated that house entry by malaria mosquitoes can be reduced substantially by elevating the house off the ground [7] and by increasing ventilation to reduce indoor levels of carbon dioxide [8], making it difficult for mosquitoes to locate a blood meal and transmit malaria. Indoor air pollution mostly due to smoke from open fires but also due to dust and allergens from earth floors, muddy walls, and thatched roofs increases the risk of respiratory tract infections [9, 10]. Poor water supply and sanitation predispose to diarrhoeal diseases [11–13].

The novel house design evaluated in this study addresses these environmental health risks. The underlying concept for the design is derived from traditional rural Asian house designs, which are elevated and constructed of light, air-permeable materials that facilitate airflow to minimize contact with vectors [3]. A pilot study of six prototype houses carried out in Magoda, Tanzania, in 2015 found that mosquito entry was reduced by 95% in double-storey buildings and by 70% in screened single-storey buildings compared with unmodified reference houses [14]. Both single- and double-storey houses were 2.3 °C (95% CI 2.2–2.4) cooler compared with traditional houses. The observed reduction in mosquito densities and temperature suggest that double-storey screened houses could protect best against malaria by providing a barrier to mosquito entry and encouraging bednet use in the cooler indoor environment. By promoting airflow and installing chimneys that reduce indoor smoke inhalation, the risk for respiratory tract infections could be reduced. Rainwater collection systems and hygienic, fly-proof latrines could reduce the risk of diarrhoeal disease transmission.

## Study objectives

### Primary objective

The primary clinical objective of the study is to assess over a follow-up period of 3 years whether living in a novel design house will reduce the incidence of malaria in children compared to children living in traditional sub-Saharan African homes.

### Secondary objectives

The secondary clinical objectives of the study are as follows:

- To determine whether living in novel design houses reduces the incidence of respiratory tract infections in children
- To determine whether living in novel design houses reduces the incidence of diarrhoea diseases in children
- To determine whether living in novel design houses reduces *Plasmodium falciparum* parasite rates in children
- To assess whether living in novel design houses results in improved childhood growth by reducing the incidence of illness
- To determine whether living in novel design houses decreases overall childhood hospitalization rates
- To identify and compare the predominant pathogens causing respiratory tract and diarrhoeal diseases between the study arms
- To compare the numbers of malaria mosquitoes and domestic flies entering novel design and traditional houses
- To assess the use and acceptability of the novel design houses
- To compare the frequency of ITN use is both study arms
- To assess the potential cost-effectiveness of the novel design house
- To compare indoor temperature, relative humidity, carbon dioxide, and atmospheric particulate matter with a diameter of less than 2.5 μm (PM_2.5_) concentrations in both study arms
- To compare the relative aerosol dispersion rates between the study arms
- To assess the durability of the various components of the novel design house

## Methods and study design

### Study area

The study site is in the Mtwara region, on the southeast coast of Tanzania, which shares its southern border with Mozambique. It is an area of forest and scrubland with a very high population-adjusted *P. falciparum* parasite rate standardized to the age group 2–10 years (PAPfPR 2–10 > 30%) [15]. Historically, the climate consists of two rainy seasons: the long rains between February and April and the short rains from late October to December. The lush, green vegetation in the area is replaced by a dry, barren landscape between the rains. Mtwara has an area of 16,710 km^2^ and comprised five districts and nine councils with a population of 1,424,083 people in 2018 [16]. Mtwara region is a leading cashew producer in Tanzania, and more than 90% of its residents are engaged in cashew production, with a small number of fishers [17]. The study will be conducted in 60 villages with households that meet the inclusion criteria of the study (see below) randomly selected from Mtwara’s 110 villages. The study population has no prior experience with health research.

### Study design

This is an open-label, household-cluster, randomized, controlled, superiority trial using a generalized, randomized, complete, block design, with the village as a block. The study arms consist of 110 novel-design houses and 440 traditional African homes.

### Intervention

**Fig. 1:**
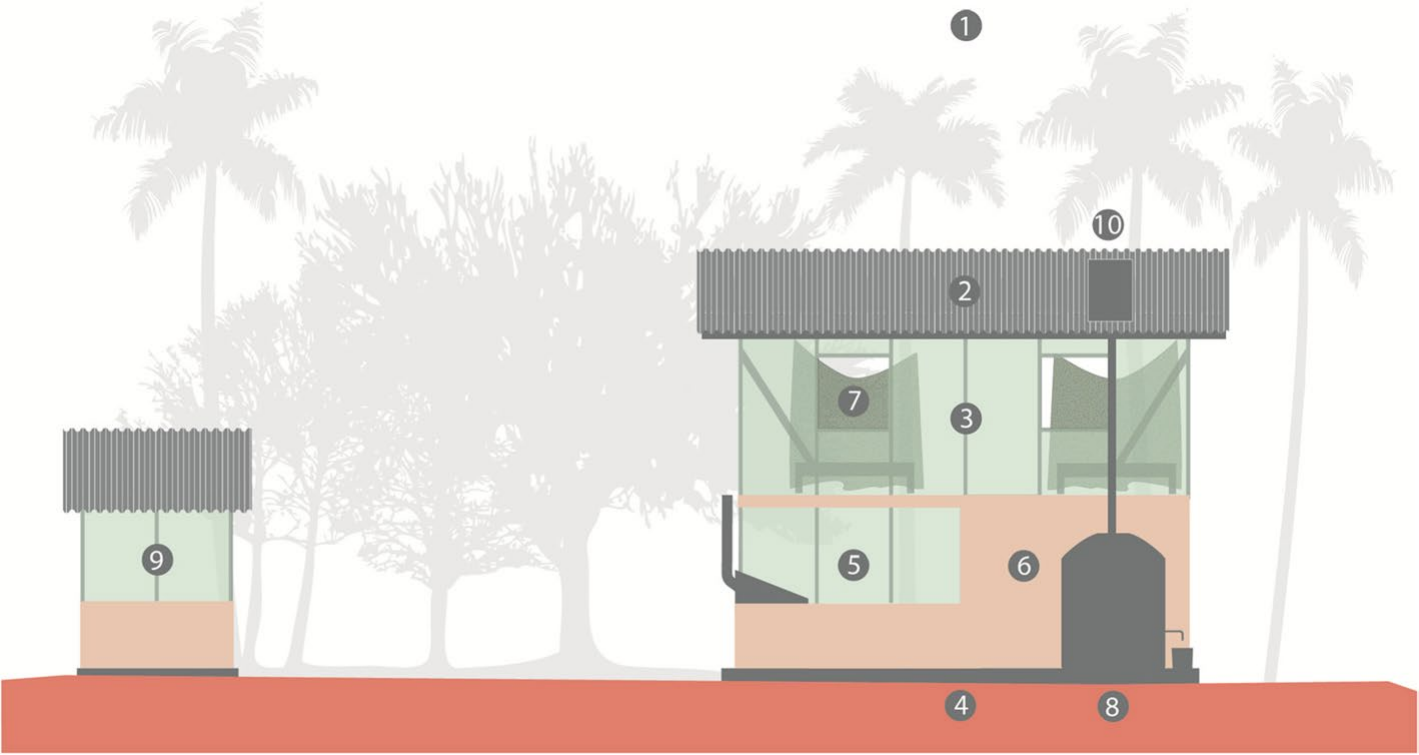
Diagram illustrating the critical elements of the novel rural African house design

The intervention consists of a novel house design. Its structure is informed by pilot studies in Magoda, Tanzania [14]. The critical components are an elevation of the sleeping area by constructing double-storey buildings and the use of shade net as cladding to optimize airflow while minimizing the entry of insects (Fig. 1). To assure timeliness in construction and identical physical characteristics of all new houses, a light gauge steel structure was chosen by the architects. The building site was selected by the house owners in discussion with the architects to address the requirements of the house owners and optimize shade throughout the day. The raised concrete foundation serves as the ground floor and can be easily cleaned. The ground floor includes a protected lockable storage area to reduce rodent infestation and to provide a feeling of security. Each of the 3 doors comes with a spring-powered closing mechanism to minimize the time that the doors stay open. The frequency of opening and closing of the door will be recorded by data loggers via sensors. The ground floor cooking area is screened to improve airflow and reduce the entry of flies. The kitchen includes a chimney to direct the flow of smoke away from the building. Rainwater is harvested in roof gutters, which drain into 2000-l PVC water tanks. The outdoor latrines are constructed within a 10-m radius of the building, using a light gauge steel system with a fly-proof, ventilated septic tank. Each house comes with a solar power system which provides electricity for light and mobile phone charging. The new homes are designed to minimize energy consumption through reduced use of concrete and optimized airflow and ventilation. By our estimates, a novel design house uses 37% less embodied carbon, 41% less embodied energy, and 70% less concrete than a more traditional African home design of similar size [18].

One of the requirements for a family to participate in the lottery for a house was to have space to build a new house. This building site on the land available to the future house owner was selected in discussion between the house owner and the architects taking into account any slope in the terrain and trees which can provide shade. The orientation of the building in relation to the north-south axis was determined by the architects. The ultimate position for the latrine was the result of the discussions between the house owners and the architects. Mosquito breeding sites of mosquitoes were not taken into consideration. Because the space available for construction was limited, the new house frequently replaced the earlier traditional home.

The construction of the 110 novel-design houses started at the end of 2018 and was completed in June 2021 when the houses were handed over to the new house owners. A maximum of three new houses were built in each village to minimize the risk of mosquito diversion, i.e. mosquitoes prevented from entering an intervention house being diverted to a control house and thus inflating malaria transmission in control houses. The residual mosquito diversion is unlikely to increase exposure in unprotected homes [19].

To reduce malaria and enteric and respiratory tract infections and to improve the indoor climate and safety of our latest design (Fig. 1), the following critical structural components are included:

1. Building orientated to provide optimal shading throughout the day to keep the house cooler at night (→ indoor climate)
2. Lightweight and durable roof with partially closed eaves to reduce the entry of malaria vectors (→ malaria, indoor climate)
3. Facade and openings screened to reduce insect entry while assuring airflow (→ malaria, diarrhoeal diseases, indoor climate)
4. Raised concrete ground floor which can be easily cleaned and increases hygiene and reduces the risk of enteric and soil-transmitted infections (→ diarrhoeal diseases)
5. A screened indoor cooking area with the means to remove smoke to reduce indoor pollution (→ respiratory infections)
6. A protected lockable storage area to reduce rodent infestations and provide a feeling of security (→ safety)
7. Sleeping areas with bednets raised to the first floor improving airflow and comfort while reducing mosquito density (→ malaria, indoor climate)
8. A water harvesting system which allows the collection of rainwater from the roof, filtering, and covered storage (→ diarrhoeal diseases)
9. An outdoor fly-proof latrine (→ diarrhoeal diseases)
10. Solar power providing electric light at night (→ safety)

#### Participant eligibility

The randomization procedure started by identifying suitable villages based on the eligibility criteria described below.

The following are the village eligibility criteria:

- Size: a village of about 500 people or more (about 100 households), to make sure that we would have at least five households for randomization.
- Accessibility: readily accessible throughout the year to assist during the construction phase and surveillance.
- No competing health interventions: we selected a study area without plans for new, experimental health interventions. The routine government health interventions including the distribution of ITNs continues.
- No power supply: lack of electricity is representative of most rural Tanzanian villages.
- No water supply: few rural Tanzanian villages have access to reliable water supplies.

Following the selection of 68 eligible villages, a census of 14,600 households in the 68 villages was conducted, and data on household members and household structures were collected [17]. Data were entered in tablets and subsequently uploaded, stored, and processed using the KoBo Toolbox. Eligible households were selected according to the criteria below.

The following are the household eligibility criteria:

- There is sufficient land on which to construct another house.
- At least two children under 13 years old (no lower age limit).
- Willing to participate in disease surveillance for 3 years.
- Traditional house design constructed with mud walls, thatched roof, and dirt floor.
- Householders had access to a pit latrine.
- No power supply from the grid.
- No piped water supplied by the municipality.

Eligible households were identified in 60 villages; therefore, participants in 60 out of 68 villages participate in the study.

### Randomization

**Fig. 2:**
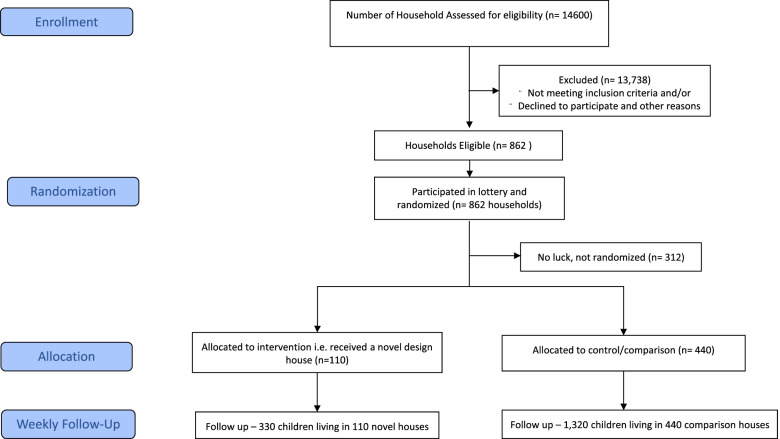
Assembly of study households and participants

All households meeting the eligibility criteria were invited to participate in a randomization process in the form of a village lottery. A total of 862 households were eligible to participate in a lottery to select firstly the 110 new house owners and secondly the 440 households to serve as comparison houses. The lottery was conducted during a public meeting at a central meeting place to which all villagers were invited. All households meeting the inclusion criteria were listed, and cards with the names of the household’s heads were inserted in identical envelopes. The envelopes were placed in a transparent bucket and mixed. The lottery consisted of two rounds where the winning envelope was picked by a literate school child of any age. In the first stage, two envelopes were picked out of the bucket, and the winners were offered a new house. During the second round of the lottery, eight households were randomly selected to participate in the study as comparison households. Comparison households will receive modifications of their homes such as a corrugate iron roof and water harvesting equipment after completion of the 3-year surveillance period. All study families, control and intervention, are enrolled in a health insurance scheme which assures free healthcare for the study duration. Each household meeting the eligibility criteria had an equal and fair chance of being selected to be part of the study (Fig. 2).

### Community engagement

A hierarchical approach to engage the study population was chosen at the earliest contact with the prospective study communities. The study team sought the advice of the district government and based on their recommendations approached the village leadership to explain the purpose and methods of the proposed study. The village leaders organized village meetings preceding census activities in the villages. Following the completion of the census, all villagers were invited to meetings to explain the purpose of the lottery described above. During the construction phase, there was regular contact with the new house owners and their neighbours. The construction team explained the activities and, where possible, asked the new house owners to participate in the construction.

Following the hand-over of the new houses, the research team realized that envy among community members who had not been allocated a novel design house was hindering study participation. In response, a multipronged community engagement plan was implemented prior to the start of active surveillance activities. A professional drama group developed plays originally meant to be enacted as village theatre. Following COVID-19-related restrictions, the script was transformed into a radio show “Samoe” with 20 episodes of 20 min each, describing the adventures of a young couple after participating in the lottery and winning a new-design home.

Due to the popularity of sports and particularly football, the research team formed a football club which participated in weekly matches in the study villages. Additional sports activities were added for all day village “bonanzas” on weekends. In conjunction with the sports days, village open-air dances were organized in-between and after sports activities. Rest periods between activities are used to introduce the research team and explain the purpose and the methods of the study.

### Clinical data collection

Each household and household members will be assigned unique study identification numbers. Clinical data will be collected at the household level from children under 13 years of age. The follow-up period is for 3 years from 2022 to 2024. Clinical data collection will comprise active and passive surveillance, annual malaria surveys and annual anthropometric surveys.

For the active case surveillance, trained research assistants will visit each child weekly and collect disease and travel history, temperature, and a rapid diagnostic test to detect *P. falciparum* infections if there is a history of fever since the last visit. A history of diarrhoea defined by 3 loose bowel movements within a 24-hour period or a bloody loose bowel movement or an acute respiratory tract infection defined as an increased respiratory rate for age and/or difficulty breathing will be elicited at each household visit. To help the family remember what happened during the week, we have designed a disease symptoms card which the head of household or any other adult in the house completes if fever, diarrhoea, or acute respiratory tract infection is observed in a study child. The research assistants will refer ill children to a healthcare facility, as needed.

Passive case detection will be conducted in all healthcare facilities serving the study population, documented in an earlier healthcare uptake/treatment-seeking behaviour study (manuscript in preparation). Study participants will be asked to identify themselves when entering the healthcare facility to benefit from the health insurance provided by the project. The staff working in the facilities will be trained on how to document the data of study participants. Research assistants will visit the health facility and electronically record the relevant information.

To detect any difference in parasite prevalence between participants in the two study arms, annual malaria surveys will be conducted. Dried blood spots (DBS) and a rapid test for malaria will be collected from all children participating in this study. This survey will be done four times throughout the 3-year study period (at the beginning, and once every year for 3 years). The DBS will be stored in freezers until processing for PCR [20, 21].

To detect any difference in growth between preschool study participants in the two study arms, anthropometric measurements (weight, height, and mid-arm circumference) will be collected at the beginning of the study and then annually.

### Microbiology

Starting in the second year of surveillance, nasal swabs will be collected from children with symptoms consistent with acute respiratory tract infections, and rectal swabs will be collected from children with diarrhoea. In addition, a swab will be collected from a child without symptoms residing in a control house. The swabs will be transported in an appropriate transport medium at ambient temperature to the study hub in Mtwara town where the specimens will be stored at − 20 °C until final processing using multiplex PCR [22]. An air sampler will be used to collect standardized air samples to detect pathogens in air samples [23].

### Entomology

Standard CDC light traps will be used to estimate the potential exposure to malaria mosquitoes in the bedrooms of study children in 110 novel design houses and their nearest comparison house from October 2021 to December 2023. The study area will be divided into seven geographically separated clusters, six each with 16 Star homes and 16 control houses and one with 14 Star homes and 14 control houses. Each cluster will further be divided into four sub-clusters, each with eight houses: four Star homes and four nearest neighbours’ control houses. The light traps will be suspended 1 m from the ground at the foot end of a bed where a study child is. Collections will be made from one cluster each week, with a different sub-cluster sampled on four consecutive nights each week, Monday night to Thursday night. At the end of the week, all houses will be sampled in each cluster (i.e. 32 houses). The sequence in which clusters will be visited was randomly determined. The clusters will be visited in the same sequence throughout the study period, so that each cluster will be visited in a 7-week cycle. Malaria vectors collected in the study houses will be counted and compared between the intervention and control houses and then packed in the Eppendorf tubes containing silica gel and submitted to the laboratory, for further analyses. The mosquitoes will be identified by microscopy and the numbers of *An. gambiae* s.l., *An. funestus* s.l., and other species will be recorded. The presence of sporozoites will be identified using an enzyme-linked immunosorbent assay [24], and *An. gambiae* s.l. females, typed by PCR [25].

The abundance and species of domestic flies will be assessed using baited traps installed in the cooking area of selected households, outdoors, and in latrines to assess the difference in fly densities between the two study arms. The traps will be positioned on the kitchen floor, in the corner furthest from the main door. Research assistants will collect the captured flies each evening.

### Social science

A mixed-method approach will be deployed for the assessment of acceptance and the use of each component of the novel design house (i.e. kitchen, storage area, bedroom, latrine, water collection facility). Data will be collected using focus group discussions (FGDs), in-depth interviews (IDIs), and a structured questionnaire-based survey at the beginning of the study and after 1 to 2 years. In-depth interviews will be conducted with approximately 18 selected heads of the households as well as selected members of the community (community leaders, health workers, religious leaders, etc.) to get their perspectives of the new house designs (design elements, construction materials, aesthetic appearance, etc.) and use of the house. FGDs will be conducted with a group of eight to 10 participants including respondents from both study arms and other selected members of the community to explore the community’s perception of the novel design houses. Approximately 10 FGDs will be conducted with approximately a total of 100 participants taking part in the discussions. The construction costs of novel design houses will be estimated and used to assess incremental costs and cost-effectiveness of the interventions.

### Architecture and environment

Data loggers will be used to record environmental data. The data will be recorded in intervals throughout the follow-up period and downloaded weekly or monthly intervals depending on the storage capacity of the device. Rainfall, temperature, relative humidity, and wind speed and direction will be recorded using an automatic weather station placed in the centre of the study area. Indoor temperature, relative humidity, and carbon dioxide concentrations will be recorded in the bedrooms of study homes in eight Star homes and eight neighbouring traditional study homes each week. PM2.5 will be monitored in the kitchens and bedrooms of study and control houses. Readings will be made every 30 min for three consecutive nights. GasLab data loggers will be positioned at the foot end of the study child’s, 1 m above the floor. Aerosol dispersion will be studied using computational fluid dynamics to digitally model the aerosol movements in space. The model will be informed by the environmental data and further qualified by targeted on-site registrations.

### Timeline for the star homes project

**Fig. 3:**
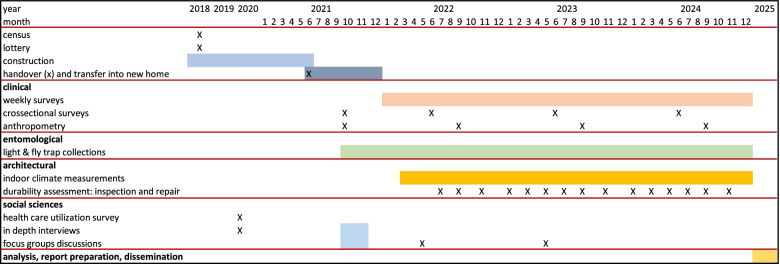
Estimated timeline for the Star Homes Project. Project activities started in 2018 and will be completed in 2025

The timeline for the star homes project is presented in Fig. 3.

### Mitigation of bias and confounding

The intervention—a novel house design—makes it impossible to conduct a blinded trial. Researchers and village residents are aware of who lives in an intervention and a control house. The awareness of the intervention allocation can lead to differences in the detection of diseases and hence introduce bias. The study team aware of this risk will take all possible steps to prevent differences in data capture between the study arms. Observer bias will be minimized wherever feasible.

Stratified randomization by village will reduce the likelihood of imbalances due to differences in geographic characteristics between the study groups. In addition, data on potential confounding factors will be collected and corrected for in the analysis. Laboratory technicians will be blinded to the identity and intervention status of the subjects. Collector bias will be reduced by using standard light traps and fly traps which do not rely on the ability of the fieldworker to collect specimens. Trap catches will be examined by a different person blinded to the trap location.

The study participants can only benefit from the house design intervention if they make use of the new houses and sleep upstairs. Children moving between houses and not regularly sleeping upstairs in study houses will be identified during weekly interviews with the household head or guardian and censored in the analysis.

### Safety and adverse events

Since the intervention, a novel design house is no health or safety risk which could expose participants to harm neither a data safety monitoring board (DSMB) has been appointed nor is an interim analysis planned. It is not reasonable to anticipate that novel design houses which have been carefully planned with regard to safety features will lead to an increase in adverse events.

Study households are visited weekly by research assistants, and structural problems such as tears in the shade net used for screening, solar electricity supply, or water harvesting are addressed and repaired within days. In the unlikely case that the study team is alerted of a safety issue, the problem will be addressed immediately by our standby construction/repair team in charge of building maintenance.

### Auditing

No audits are planned.

### Study endpoints

#### Primary endpoints

Incidence of malaria will be calculated in children under 13 years participating in the study for 3 years, using the number of detected cases as the numerator and the period contributed by each child residing in the study area as the denominator. These data will be analysed and used to determine the relative risk of malaria among children living in the novel design houses compared with those living in traditional sub-Saharan African homes.

#### Secondary endpoints

Incidence of respiratory tract infections and diarrhoea diseases will be calculated, and relative risks determined among children living in the novel design houses compared with those living in traditional African homes.

##### *P. falciparum* prevalence

The prevalence of malaria parasitaemia among children living in novel design houses and traditional homes will be compared in annual malaria surveys. Finger prick blood specimens will be collected from all children participating in the study for *P. falciparum* rapid diagnostic test and dried blood spots for qPCR.

##### Difference in growth parameters

Weight, height, body mass index (BMI), and mid-upper arm circumference of all children participating in the study will be recorded at baseline, annually, and at the end of the study. The mean changes in growth parameters will be compared among children living in intervention and control houses.

##### Difference in disease severity

Differences in disease severity between children residing in novel design houses and traditional homes will be analysed by comparing hospitalization rates.

##### Microbiology

Differences in the frequency of pathogens detected in symptomatic and healthy children will be used to calculate attributable fractions of pathogens and compared between intervention and control houses. Pathogens detected by air sampling will be compared between intervention and control houses.

##### Mean number of malaria vectors/light trap/night

The number and type of mosquitoes from novel design houses and traditional homes will be determined at various time points throughout the study period. This will be used to assess the differences and similarities in mosquito densities between novel design houses and traditional homes as a proxy for measuring exposure to malaria transmission.

##### Environmental measurements

Indoor climate and other relevant environmental metrics will be measured for 3 years using data loggers. This will be used to compare indoor temperature, humidity, carbon dioxide, PM_2.5_ particle pollution, and door opening and closing in both study arms.

##### Acceptability of interventions

The acceptance of novel design houses will be assessed using qualitative and quantitative research methods. Interviews will be conducted 6 months after moving into the houses and after 1 to 2 years.

##### Bednet use

Household heads of traditional and novel design houses will be interviewed annually to estimate the use of ITNs.

##### Durability

Yearly inspections of all study houses will be conducted to assess durability and modifications of the various components of the building structure.

##### Economics

A comparison of construction costs and cost benefits for intervention and control houses will be conducted.

##### Aerosol dispersion

The risk of becoming infected with SARS CoV-2 (COVID-19) between the intervention houses and the control houses via aerosols from another occupant of the same household, will be compared.

### Sample size calculations

**Fig. 4:**
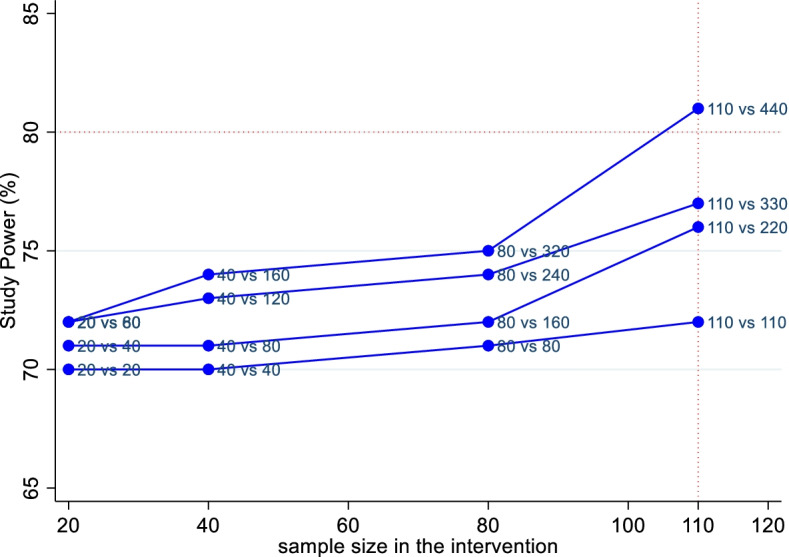
Simulated power calculations showing power by ratios of intervention to control houses, assuming a 30% reduction in all diseases under investigation

Our sample size estimates are based on malaria incidence in Korogwe, Tanzania, observed during the RTS,S cohort phase 3 trial [26]. Malaria epidemiology in Tanzania is quickly changing, and the main burden of disease has moved from young children aged 5–17 months to older children and adolescents. Assuming malaria episodes of 10/100 children/year in the control and a 30% reduction from 10 to 7 malaria episodes/100 children/year, a coefficient of variation (CV) of the outcome of 0.25, as recommended by Hayes and Moulton 2009 [27], detecting the difference in incidence rates with 80% power and testing at 5% significance level, we would need 105 clusters (households) with three child/years of observations per household per arm. To allow for loss to follow-up, we plan to recruit 110 households to the intervention arm. Since analysis requires adjustment for clustering by village and household [28], power simulations were performed using a random effects model to assess what ratio of traditional to novel-design households provides adequate power. For every intervention house, we will recruit four control houses, which will provide at least 80% power at the 5% level of significance to detect a 30% risk reduction. Since ICCs that accommodate village and household level clustering are rarely reported, we compensated for clustering from both levels by choosing a conservatively high ICC of 0.6. Because malaria is generally rarer than either respiratory diseases or diarrhoeal diseases in the study population, the sample size should suffice to measure a 30% reduction in all diseases under investigation at a 5% level of significance and 80% power. The sample size was performed in Stata 15 using the following basic command: clustersampsi, samplesize rates r1(.1) r2(.07) m(10) cluster cv(0.25) alpha(0.05) beta(0.8) base correl (0.6). The simulation results are shown in Fig. 4 below. Simulations were performed in Stata, and each scenario was simulated 5000 times and the empirical power was obtained. We considered 20, 40, 80, and 110 households in the intervention.

### Entomological outcomes

We postulate that the intervention homes will reduce indoor collections of malaria vectors by at least 50%. Based on a recent study using light traps in the Kilombero Valley, Tanzania, the mean number of female *An. gambiae* s.l. per trap in a house was 10.4 (SD = 21.5). Computer simulations based on a negative binomial distribution suggest 89% power at the 5% level of significance to detect a 50% reduction in indoor-entering mosquitoes (i.e. *An. gambiae* s.l.) 110 Star Homes and 440 control houses will be needed. We propose to collect a sample from each house every 7 weeks each year for 3 years.

### Acceptability outcomes

The total number of interviews will depend on when data saturation is reached, i.e. when it is unlikely to lead to the identification of any significant new issue or novel insight. We hope to reach saturation with 18 in-depth interviews with heads of households and 10 focus group discussions with approximately 8 to 10 participants per group.

### Ancillary and post-trial care

As mentioned earlier, all families in homes participating in the study irrespective of whether they are in the control and intervention arm are enrolled in a health insurance scheme paid for by the study which assures free healthcare for the study duration. Furthermore, the study team assures that all study participants and their families receive the promised services free of cost. Not all healthcare providers working in institutions participating in the insurance scheme are yet fully aware of the operational aspects of the insurance scheme which has only recently been introduced in Tanzania. The free insurance coverage and free house maintenance will be phased out in 2025 the year after the completion of clinical surveillance. There are reasons to hope that at least some of the study households will continue enrolment in the insurance scheme after study completion.

### Dissemination policy: trial results and authorship

The study findings will be disseminated by publication in the peer-reviewed journals and presented in scientific conferences. The findings will be shared with the study participants’ community through community meetings and summary leaflets that will be provided to the village offices. The investigators will be involved in reviewing drafts of the manuscripts, abstracts, press releases, and any other publications arising from the study. Authorship will be determined in accordance with the ICMJE guidelines and other contributors will be acknowledged.

### Availability of data and materials: reproducible research

The study data will be available upon reasonable requests to the Mahidol Oxford Tropical Medicine Research Unit Data Access Committee (http://www.tropmedres.ac/data-sharing) for researchers and following the Mahidol Oxford Tropical Medicine Research Unit data access policy (http://www.tropmedres.ac/_asset/file/datasharing-policy-v1-1.pdf).

### Analytical plan

#### Incidence of malaria, respiratory tract infections, and diarrhoea diseases

Protective efficacy against clinical malaria, pneumonia, and diarrhoea diseases will be assessed by comparing incidence rates between the two study arms adopting an intention-to-treat analysis. Children within each household will be monitored using active and passive case detection throughout the study period. After treatment for malaria, the child will not be considered at risk for the following four weeks and this period will be censored. History of travel and nights spent away from the designated household will be captured during weekly surveillance visits and time at risk will be censored for such periods. Initial unadjusted analyses will be based on the comparison of the incidence rates between the two arms. Formal analyses will use a mixed effects Poisson model to test the difference in incidence rate between the two arms allowing for the repeated measurements within a house, a village and the effect of year using of confidence intervals that account for the highest level of clustering [28]. With multiple control and intervention houses within each village, the “intervention*village” interaction will also be quantified. Possible confounders such as age of child, gender, ethnicity, and rainfall will be tested using the mixed effects model. A mixed effects negative binomial model will be considered in case of overdispersion in the mixed effects Poisson model. A comparison of time to infection (both first and repeat) will be examined using a survival analysis approach. Initial analyses will be based on Kaplan-Meier curves with further adjustment for confounders performed using a Cox regression model.

#### Entomology (malaria transmission)

Differences in malaria transmission experienced in the two groups will be made by comparing the mean number of malaria mosquitoes (as a proxy for transmission) caught indoors in houses between the study arms. Generalized estimating equations will be used to estimate the differences in numbers of indoor-resting mosquitoes, adjusting for repeated measures within houses and possible covariates. We will compare the entomological inoculation rate in both arms. Human behavioural studies will also be carried out to compare when people move indoors and outdoors of the house, since the new housing intervention may change the way individuals use a house, which changes their exposure risk. Observers will record human movement including the age and gender of individuals with their identity anonymized.

#### Clinical (*P. falciparum*, respiratory infections, and diarrhoeal diseases)

*P. falciparum* prevalence in children will be estimated at the start of the transmission season through a cross-sectional survey. Falciparum malaria and respiratory and enteric disease incidence will be estimated based on active and passive surveillance and compared between the study arms. The incidence of all-cause febrile illness during the 3-year study and age-adjusted growth rates will be estimated in children under 5 years old.

#### Acceptability

Perceptions of the novel design house owners and the general community regarding the aesthetic appearance and convenience of the houses, use of different spaces in the house, its design elements, and construction materials will be summarized to provide an overall impression of acceptance of the novel design houses. The proportion of owners of control houses who choose to have home improvements at the end of the study will be determined.

#### ITN use

The proportion of children sleeping under ITNs between study arms at different times of the year will be compared.

#### Architectural

Indoor temperatures, humidity, CO_2_, and PM_2.5_ particle pollutions will be quantified using data loggers and compared between the two study arms. This will provide data regarding the level of air pollution between novel design and traditional homes and whether it correlates with respiratory tract infections. The durability of the novel design houses will be assessed by yearly inspections of houses. In traditional houses, maintenance records will be kept to allow comparisons between the study arms.

#### Aerosol dispersion

*Aerosol dispersion* will be modelled using computational fluid dynamics (CFD). The CFD simulations will provide insights into the effect of various environmental parameters on the transport/dispersion of aerosols between indoors and outdoors in novel design houses and traditional houses, respectively, and subsequently allow us to assess the relative risk of airborne transmission of COVID-19 between inside and outside of a home.

## Discussion

The idea that better house design leads to better health is for most readers intuitively convincing. Evidence in support of this notion is however scant and generally considered of questionable quality because it is not derived from randomized controlled trials [29]. The rapid growth of a population in Africa provides an opportunity for research on specific house design elements that are associated with health benefits. From 2000 to 2015, the prevalence of homes in sub-Saharan Africa with improved water and sanitation, and of durable construction doubled from 11 to 23% of houses [30]. The results from this study could potentially influence the design of future housing in sub-Saharan Africa.

A systematic review of 90 studies published in 2015 found overwhelming evidence that house improvements, described as “modern houses”, have a protective effect against malaria [31]. Four randomized controlled trials of house screening have been published, with three trials demonstrating protection against malaria [32–34] and one outlier [35].

Whether housing improvements can protect against respiratory tract infections and diarrhoea is less obvious. A systematic review published in 2021 “indicated a potential benefit of home environmental interventions in preventing overall respiratory tract infections” [36]. In contrast, a “big data” approach re-analysing diverse surveys that included more than 800,000 children found that housing improvements reduced the odds of childhood malaria and diarrhoea but failed to detect any protection against acute respiratory tract infections [37]. Considering the disparity of interventions and geography, this systematic review may not be ideal to predict whether novel house designs can protect against respiratory tract infections in rural Tanzania. Similarly, re-analysing a range of surveys conducted for purposes other than the assessment of health benefits of housing improvements may neither be specific enough to accurately distinguish relevant housing improvements nor reliable enough to detect acute respiratory tract infections.

The strategies for the prevention of diarrhoea include the provision of safe drinking water, sanitation, and hygiene (WASH), all of which can be addressed by home improvements. Yet, the evidence of whether WASH strategies can indeed prevent diarrhoea and thereby improve childhood growth is at best equivocal. Three large, recent trials [38–40] of unprecedented scale and cost found mixed effects on diarrhoea and no protection against stunting [41]. We hypothesize that controlling multiple childhood diseases will have multiplicative health benefits. If correct, we anticipate less severe disease and less cumulative uncomplicated disease will ultimately result in less malnutrition and better growth parameters of children living in novel houses compared with those living in traditional houses.

The majority of homes currently constructed in sub-Saharan Africa have concrete floors, concrete walls, and a corrugate iron roof. A house design pattern emerging throughout sub-Saharan Africa is reminiscent of housing developments in the US or Europe but is heavily dependent on air-conditioning to maintain a comfortable indoor climate in a hot tropical climate. Cement production is highly energy-intensive as is air conditioning [42]. The design of the houses in the study is a notable exception from this trend since the use of concrete and the need for air-conditioning are intentionally minimized.

Although unique and without precedent our study has several limitations. Firstly, it is impossible to blind the study subjects and field researchers from the intervention. We will try to address this handicap by conscientiously assuring that the same procedures are applied to all participants and that those analysing samples in the laboratory are blinded to the source of the sample. Secondly, since our primary outcome is disease incidence, the capture of all disease episodes is of critical importance. This will be achieved through a combination of active cases detection supported by passive case detection in all health facilities in the study catchment area. It is possible to miss disease episodes if the guardians of study participants fail to inform the research assistants and seek either no healthcare at all or seek healthcare outside the recognized system. Building rapport and ultimately gaining the trust of the families living in study houses will be the key strategy for the capture of all disease episodes. Thirdly, some families are sceptical about using the novel houses. Community sensitization and engagement will continue throughout the trial period to ensure full compliance. A multipronged community engagement programme is underway addressing the fears of using the houses to facilitate the understanding between the study communities and the researchers. Finally, and perhaps most importantly, the interventions can only work if the study participants sleep and more broadly live in their assigned homes. If children assigned to novel design homes sleep in the homes of neighbours or relatives, they cannot be protected by the novel home design. Data will hence be collected on household use to assess how often children sleep in the novel design houses. This type of dilution of protection may be most intense for respiratory tract infections and diarrhoea where study participants can be infected at any time of the day including while attending schools without a safe water supply, and where sanitation and ventilation have not been improved. As falciparum malaria is predominantly transmitted by endophagic and endophilic vectors in sub-Saharan Africa, most of the transmission occurs at night at home. Hence the impact on malaria transmission of novel home designs may be least diluted. Potential protection against respiratory tract infections and diarrhoea could be reduced through infections acquired outside the home.

## Conclusions

This protocol describes an open-label, household randomized controlled trial to assess the protective efficacy of a novel design house against three major childhood diseases, malaria, respiratory tract infections, and diarrhoea diseases compared to those living in traditional homes. This trial provides an opportunity to collect high-grade evidence for the benefits of a range of housing interventions. The trial comes at a unique moment of what could be the onset of an intense population growth phase on the African continent. Providing millions of air conditioning units is neither feasible nor sustainable. People need comfortable and healthy homes and rapid population growth will result in massive formal and informal housing construction. Making relatively small modifications in the design of houses to reduce the risk of mosquito entry, increase ventilation, improve air quality, and assure safe water supply and sanitation could result in long-term health benefits affecting the present and future generations.

Protocol version: 1 June 2020, version 2.0

## Trial status

The random allocation of houses was completed in 2018. The house construction was completed by June 2021. The houses were handed over, and the new residents moved in between June 2021 and January 2021. The disease surveillance started in January 2022. The surveillance will be completed in December 2024. The analysis and publication of the most pertinent findings should be completed by December 2025.

## Abbreviations

ITNs: Insecticide-treated nets
CI: Confidence interval
PM2.5: Atmospheric particulate matter (PM) with a diameter of less than 2.5 μm
PAPfPR: Population-adjusted *Plasmodium falciparum* parasite rate
Fig.: Figure
DBS: Dried blood spot
PCR: Polymerase chain reaction
CDC: Centres for Disease Control
*An.*: *Anopheles*
FGD: Focus group discussion
IDI: In-depth interview
CO_2_: Carbon dioxide
CFD: Computational fluid dynamics
