# Correction: Assessing the impact of a novel house design on the incidence of malaria in children in rural Africa: study protocol for a household-cluster randomized controlled superiority trial

**DOI:** 10.1186/s13063-022-06501-8

**Published:** 2022-07-07

**Authors:** Salum Mshamu, Arnold Mmbando, Judith Meta, John Bradley, Thomas Chevalier Bøjstrup, Nicholas P. J. Day, Mavuto Mukaka, Fredros Okumu, Ally Olotu, Christopher Pell, Jacqueline Deen, Jakob Knudsen, Steven W. Lindsay, Lorenz von Seidlein

**Affiliations:** 1 CSK Research Solutions, Mtwara, Tanzania; 2 Nufeld Department of Clinical Medicine, University of Oxford, Oxford, UK; 3 Ifakara Health Institute, Ifakara, Tanzania; 4 Department of Biosciences, Durham University, Durham, UK; 5 University of Amsterdam, Amsterdam, Netherlands; 6 MRC International Statistics and Epidemiology Group, London School of Hygiene and Tropical Medicine, London, UK; 7 The Royal Danish Academy of Fine Arts, Copenhagen, Denmark; 8 Mahidol-Oxford Tropical Medicine Research Unit (MORU), Bangkok, Thailand; 9 University of Philippines, Manila, Philippines

**Correction: Trials 23, 519 (2022)**

**https://doi.org/10.1186/s13063-022-06461-z**

Following the publication of the original article [1], we were notified that the name of the 5^th^ author was incorrectly spelled as Chavalier instead of Chevalier.

Also, January 2021 should be January 2022 in Trial status on page 12: “The random allocation of houses was completed in 2018. The house construction was completed by June 2021. The houses were handed over, and the new residents moved in between June 2021 and **January 2022**.”

The original article has been corrected.
